# Anaemia is not a risk factor for progression of acute kidney injury: a retrospective analysis

**DOI:** 10.1186/s13054-016-1231-7

**Published:** 2016-03-08

**Authors:** Jonah Powell-Tuck, Siobhan Crichton, Mario Raimundo, Luigi Camporota, Duncan Wyncoll, Marlies Ostermann

**Affiliations:** Department of Critical Care, Guy’s & St Thomas’ Foundation Hospital, London, UK; Division of Health and Social Care Research, King’s College London, London, UK; Hospital de Santa Maria, Centro Hospitalar Lisboa Norte, Lisbon, Portugal; Department of Critical Care, King’s College London, Guy’s & St Thomas’ Foundation Hospital, London, UK

**Keywords:** Acute kidney injury, Haemoglobin, Anaemia, Renal recovery

## Abstract

**Background:**

In hospitalised patients, anaemia increases the risk of developing acute kidney injury (AKI). Our aim was to determine whether anaemia also has an impact on the risk of progression from early AKI to more severe AKI in critically ill patients.

**Methods:**

We retrospectively analysed the data of patients admitted to the adult intensive care unit between 2007 and 2009 who had AKI I as per the AKI Network classification, and who had undergone haemodynamic monitoring within 12 h of AKI I. We collected baseline characteristics, severity of illness, haemoglobin (Hb), and haemodynamic parameters in the first 12 h of AKI I and differentiated between patients who progressed to AKI III and those who did not. Univariate and multivariate logistic regression analyses were used to identify risk factors for progression. Associations between Hb, arterial oxygen saturation and cardiac index were explored by receiver operating characteristic curve analysis.

**Results:**

Two hundred and ten patients (median age 70 years, 68 % male) underwent haemodynamic monitoring within 12 h of AKI I; 85 (41.5 %) progressed to AKI III. The proportion of patients with underlying cardiac disease was significantly higher among progressors versus non-progressors (58 % vs 34 %, respectively; *p* = 0.001). On the first day of AKI I, progressors had a significantly higher Sequential Organ Failure Assessment score (9 vs 8; *p* < 0.001), lower cardiac index (median 2.6 vs 3.3 L/min/m^2^; *p* < 0.001), higher arterial lactate (2 vs 1.6 mmol/L; *p* < 0.001), higher central venous pressure (16 vs 13; *p* = 0.02), lower mean arterial blood pressure (median 71 vs 74 mmHg; *p* = 0.01) and significantly higher requirement for cardiovascular and respiratory support, but there was no difference in Hb concentration (median 96 g/L in both groups). Multivariable regression analysis showed that heart disease, need for mechanical ventilation, arterial lactate, Sequential Organ Failure Assessment score, central venous pressure and cardiac index on first day of AKI I were independently associated with progression to AKI III. There was no significant difference in the risk of progression between patients with Hb ≤ or >80 g/L, and ≤ or >100 g/L on day of AKI I.

**Conclusions:**

In critically ill patients with AKI stage 1, anaemia was not associated with an increased risk of progression to more severe AKI.

## Background

Acute kidney injury (AKI) is a common complication of critical illness, affecting more than 50 % of patients admitted to intensive care units (ICUs) [1, 2]. There is a clear association between severity of AKI and risk of short and long-term complications, including death and development of chronic kidney disease [3, 4]. Management of AKI is supportive with focus on correction of volume depletion, haemodynamic optimisation and avoidance of further nephrotoxic insults. It is advised that management is tailored to AKI stage, including early use of haemodynamic monitoring [5], with the aims to reverse the injurious process within the kidneys, to induce renal recovery and to prevent progression to severe AKI.

We recently reported that a lower mean arterial pressure (MAP), reduced systemic oxygen delivery and increased fluid administration were independently associated with a higher risk of progression from AKI stage I to AKI stage III [6, 7]. However, it is unknown whether anaemia is also a risk factor. This is an important question for several reasons, since:

1. Anaemia is very common during critical illness and blood transfusions are often withheld until haemoglobin (Hb) is reduced to <70–80 g/L based on large randomised controlled trials which showed no survival benefit in patients with a higher Hb [8–10].
2. Hb is a key contributor to oxygen delivery.
3. Anaemia is a risk factor for the development of AKI in hospitalised patients [11], after major surgery [12–17] and following cardiac procedures [18, 19].
4. In patients undergoing cardiopulmonary bypass surgery, haemodilution to a haematocrit of <24 % is associated with an increased risk of postoperative AKI [20].
5. In patients with chronic kidney disease (CKD), correction of anaemia has been shown to slow the progression of renal failure [21, 22].
6. In patients undergoing cardiac surgery, a single-dose erythropoietin (EPO) administered before surgery resulted in significant reduction in postoperative AKI [23, 24].

The aim of this study was to determine whether anaemia has an impact on the risk of progression from early AKI to more severe AKI during critical illness. We hypothesised that critically ill patients with AKI and a low Hb had a reduced chance of renal recovery compared to AKI patients with a higher Hb.

## Methods

We retrospectively reviewed an electronic database (CareVue, Philips) containing data from all patients admitted to Guy’s & St Thomas’ NHS Trust ICU between July 2007 and June 2009, and identified those with AKI stage I as per serum creatinine criteria of the AKI Network (AKIN) classification [25] in whom advanced haemodynamic monitoring had been initiated for clinical reasons within 12 h of the patient meeting the criteria for AKI stage I. Renal transplant patients, re-admissions and patients who left the ICU within 24 h of diagnosis of AKI I or developed AKI stage III within 12 h of diagnosis of AKI I were excluded. The outcome of interest was progression to AKI stage III as defined by the AKIN classification, i.e. rise of serum creatinine greater than threefold from baseline or to ≥354 μmol/L with an acute rise of ≥44 μmol/L, or requirement for renal replacement therapy (RRT) [25]. The methods have been described in more detail elsewhere [6, 7].

### Data collection

We recorded demographics, comorbidities, diagnosis, Acute Physiology and Chronic Health Evaluation (APACHE) II score and Sequential Organ Failure Assessment (SOFA) score on admission to ICU and first available cardiac index, contemporaneous arterial oxygen saturation (SaO_2_), Hb, arterial lactate, central venous pressure (CVP), MAP and urine output during the first 12 h period after diagnosis of AKI I. We also recorded SOFA score, 24-h urine output, cumulative fluid balance, need for mechanical ventilation and cardiovascular support, and presence of sepsis (as per current consensus criteria [26]) on day of AKI I. Systemic oxygen delivery index (DO_2_I) was calculated as DO_2_I = 1.34 × Hb × SaO_2_ × cardiac index.

### Statistical analysis

Categorical variables were summarised as frequency (percentage). SOFA scores were summarised as mean and standard deviation (SD) and all other continuous variables, which were not normally distributed, summarised as median and interquartile range (IQR). Characteristics were compared between patients who did and did not progress using Chi-square, Fisher’s exact, Mann-Whitney or *t*-tests, as appropriate.

Associations between components of the DO_2_I (Hb, SaO_2_ and cardiac index) were explored using receiver operating characteristic (ROC) curves. The area under the curve (AUC) was used to compare the ability of the three parameters to predict which patients progressed.

To determine which components of DO_2_I (i.e. Hb, SaO_2_ and cardiac index) were most strongly associated with progression, multivariable logistic regression models were used. In addition to the three parameters, any factors which were found to be significant in univariate analyses (mechanical ventilation, coronary artery disease (CAD)/congestive cardiac failure (CCF), lactate, SOFA score on day of AKI I, CVP and mean MAP in the 12-h period after diagnosis of AKI, urine output) were also included in the model. In additional analyses, sepsis on day of AKI I was added to the model and tests for interactions between sepsis and CAD/CCF and Hb, SaO_2_ and cardiac index were performed to determine whether there were differences in risk of progression in patients with CAD/CCF or sepsis.

The sensitivity and specificity associated with cut-off points of Hb above and below 80 and 100 g/L were also calculated. The differences in odds of progression for patients in the two groups were compared using logistic regression models.

### Ethics

The study had institutional approval by the Clinical Governance Department in the hospital. As per Governance Arrangements for Research Ethics Committees published by the UK Health Departments, formal review by a Research Ethics Committee and need for individual informed consent was not required since the research was limited to secondary use of information previously collected in the course of normal care and the patients were not identifiable to the research team carrying out the research [27].

## Results

Of 2118 patients admitted to the ICU in the 2-year period, 790 (37 %) had AKI stage I; 69 patients had exclusion criteria and a further 511 patients were excluded because they did not have haemodynamic monitoring performed within the first 12 h of AKI I. The remaining 210 patients were included in the analysis (Table 1). The median number of days between ICU admission and AKI I was 1 (IQR 0–22).

**Table 1:** Baseline characteristics of patients with AKI I

Parameter	Total (*n* = 210)
Age, median (IQR)	70 (57–77)
Male gender, n (%)	142 (68)
Comorbidities	
CAD/CCF, n (%)	92 (44)
Chronic hypertension, n (%)	81 (39)
Diabetes mellitus, n (%)	40 (19)
Malignancy, n (%)	28 (13)
COPD, n (%)	27 (13)
CKD, n (%)	25 (12)
Neurological disease, n (%)	23 (11)
Chronic liver disease, n (%)	12 (6)
Admission diagnosis	
Post-surgery, n (%)	72 (34)
Cardiac, n (%)	54 (26)
Sepsis, n (%)	38 (18)
Respiratory, n (%)	34 (16)
Other, n (%)	12 (6)
Severity of illness on admission to ICU	
APACHE II score, median (IQR)	18 (14–22)
SOFA score, mean (SD)	7.1 (2.8)

AKI acute kidney injury, *APACHE* Acute Physiology and Chronic Health Evaluation, *CAD* coronary artery disease, *CCF* congestive cardiac failure, *CKD* chronic kidney disease, *COPD* chronic obstructive pulmonary disease, *ICU* intensive care unit, *IQR* interquartile range, *SD* standard deviation, *SOFA* Sequential Organ Failure Assessment

Of 85 patients (41 %) who progressed to AKI III, 78 (92 %) received RRT. The mean duration between AKI I and AKI III was 2.6 days (SD 3.4); the median duration was 2 days (IQR 1–2). Five patients with AKI I died before their renal function recovered or progressed. The remaining 120 patients with AKI I recovered renal function.

### Risk factors for progression to AKI stage III

Patients with AKI I who progressed to AKI III were sicker on the day of AKI I as evidenced by a higher SOFA score, higher arterial lactate level, lower MAP and greater need for respiratory and cardiovascular support (Table 2). There was no difference in the Hb concentration of those who progressed to AKI III and those who did not progress. Multivariable regression analysis showed that underlying heart disease (CAD and/or CCF), need for mechanical ventilation, arterial lactate, SOFA score and CVP on day of AKI I were independent risk factors for progression from AKI I to AKI III (Table 3). A higher cardiac index was independently associated with a reduced risk.

**Table 2:** Comparison of AKI progressors and non-progressors

Parameters	All (*n* = 210)*	Did not progress to AKI III (*n* = 120)	Progressed to AKI III (*n* = 85)	*p* value
Age, median (IQR)	70.5 (57–77)	70 (54–76)	71 (60–78)	0.13
Male gender, n (%)	138 (67.3)	79 (65.8)	59 (69.4)	0.59
CAD/CCF, n (%)	92 (44)	41 (34.2)	49 (57.7)	0.001
Severity of illness on admission to ICU				
SOFA score, mean (SD)	7.1 (2.8)	6.9 (2.7)	7.5 (2.9)	0.14
APACHE II score, median (IQR)	18 (14–22)	17 (13–21)	18 (15–22)	0.09
Parameters on day of AKI I				
SOFA score, mean (SD)	8.7 (2.7)	8.0 (2.5)	9.6 (2.8)	<0.001
Presence of sepsis, n (%)	125 (60)	79 (66.4)	43 (50.6)	0.02
DO_2_I (ml/min/m^2^), median (IQR)	362 (277–482)	405 (302–514)	325 (266–401)	<0.001
SaO_2_	0.95 (0.94–0.96)	0.95 (0.94–0.96)	0.95 (0.94–0.96)	0.81
Hb (g/L), median (IQR)	96 (88–105)	96 (90–105)	96 (85–105)	0.64
Cardiac index (L/min/m^2^), median (IQR)	3.0 (2.3–3.9)	3.3 (2.5–4.2)	2.6 (2.1–3.3)	<0.001
Arterial lactate (mmol/L), median (IQR)	1.8 (1.3–2.6)	1.6 (1.1–2.3)	2 (1.5–3)	<0.001
CVP (cmH_2_O), median (IQR)	14 (10–18)	13 (10–17)	16 (11–19)	0.02
MAP (mmHg), median (IQR)	73 (69–78)	74 (70–79)	71 (68–77)	0.01
Vasopressor/inotrope therapy, n (%)	187 (8)	100 (83.3)	82 (96.5)	0.003
Mechanical ventilation, n (%)	191 (91)	103 (85.8)	84 (98.8)	0.001
Urine output (mL/h), median (IQR)	61 (38–94)	66 (46–104)	54 (32–79)	0.002
Cumulative fluid balance (ml), median (IQR)	2361 (952–3812)	2379 (935–4222)	2363 (974–3719)	0.995
Outcome				
ICU mortality, %	32.4	11.7	57.6	<0.001
Hospital mortality, %	43.3	24.2	67.1	<0.001

*Including 5 patients who died before their renal function recovered or progressed

*AKI* acute kidney injury, *APACHE* Acute Physiology and Chronic Health Evaluation, *CAD* coronary artery disease, *CCF* congestive cardiac failure, *CVP* central venous pressure, *DO*_*2*_ *I* systemic oxygen delivery index, *ICU* intensive care unit, *IQR* interquartile range, *Hb* haemoglobin, *MAP* mean arterial pressure, *SaO*_*2*_ arterial oxygen saturation, *SD* standard deviation, *SOFA* Sequential Organ Failure Assessment

**Table 3:** Multivariable logistic regression models for progression

Parameters on day of AKI I	OR	95 % CI	*p* value
Mechanical ventilation	22.12	2.40–205.52	0.006
CAD/CCF	3.17	1.55–6.49	0.002
Arterial lactate (mmol/L)	1.57	1.15–2.13	0.004
SOFA score	1.21	1.04–1.39	0.01
CVP (cmH_2_O)	1.07	1.01–1.14	0.02
Cardiac index (L/min/m^2^)	0.69	0.50–0.96	0.03
MAP (mmHg) during 12 h post-AKI I	0.97	0.92–1.02	0.18
Hb (g/L)	0.82	0.65–1.03	0.09
SaO_2_	0.97	0.79–1.20	0.79

*AKI* acute kidney injury, *CAD* coronary artery disease, *CCF* congestive cardiac failure, *CI* confidence interval, *CVP* central venous pressure, *Hb* haemoglobin, *MAP* mean arterial pressure, *OR* odds ratio, *SaO*_*2*_ arterial oxygen saturation, *SOFA* Sequential Organ Failure Assessment

### Impact of anaemia

**Fig. 1:**
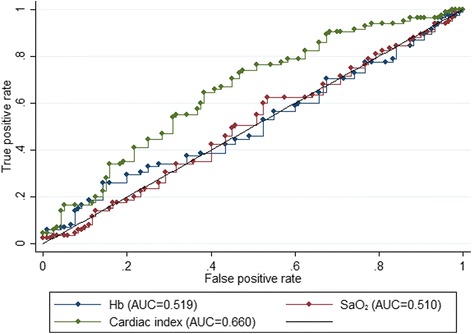
Receiver operating characteristic curves for progression to AKI stage III. *AUC* area under the curve, *Hb* haemoglobin, *SaO*_*2*_ arterial oxygen saturation

Figure 1 shows the ROC curves summarising the association between Hb, SaO_2_, cardiac index and progression to AKI III. Only cardiac index appeared to differentiate between progressors and non-progressors. There was no particular cut-off for Hb for progression. When choosing 80 g/L and 100 g/L as clinically meaningful cut-offs, there was no significant difference in the risk of progression to AKI stage III between patients with Hb <80 g/L on day of AKI I and those with a higher Hb or patients with a Hb below or above 100 g/L (Table 4). There was also no difference in the impact of Hb levels on progression in patients with or without underlying heart disease (CAD and/or CCF) and between patients with or without sepsis.

**Table 4:** Impact of different haemoglobin cut-offs on day of AKI I on risk of progression to AKI III

Hb on day of AKI I	n	Progression to AKI III, n (%)	Sensitivity	Specificity	OR*	95 % CI	*p* value
>80 g/L	190	78 (41.0)	8.2 %	93.3 %	1		
≤80 g/L	15	7 (46.7)			2.05	0.58–7.12	0.27
>100 g/L	134	55 (41.0)	64.7 %	34.2 %	1		
≤100 g/L	71	30 (42.3)			1.40	0.70–2.83	0.35

*Adjusted for arterial oxygen saturation, cardiac index, mechanical ventilation, coronary artery disease/congestive cardiac failure, arterial lactate, Sequential Organ Failure Assessment score, central venous pressure and mean arterial pressure

*AKI* acute kidney injury, *CI* confidence interval, *Hb* haemoglobin, *OR* odds ratio

## Discussion

We previously demonstrated that a higher systemic DO_2_I was independently associated with a reduced risk of progression from AKI stage I to AKI stage III. In this study we analysed the components of DO_2_I individually and found that haemoglobin concentration was not independently associated with AKI progression. Instead, the need for mechanical ventilation, the presence of underlying cardiac disease, raised lactate, higher SOFA score and a higher CVP were all independent risk factors for progression; a higher cardiac index was independently associated with a reduced risk of progression.

The relationship between anaemia and AKI is not fully understood. It is well known that AKI can contribute to the development of anaemia as a result of reduced EPO production, an increased risk of bleeding and reduced red cell life span [28]. It has also been demonstrated that anaemia is a risk factor for the development of AKI in patients undergoing major surgery leading to increased mortality [12, 14, 15]. However, it is not always clear whether the presence of anaemia is simply a reflection of co-morbid disease that increases the risk of AKI or a direct contributor to AKI, for instance as a result of anaemia-induced hypoxia in the renal cortex. The kidney, particularly the proximal tubule, is known to be very susceptible to ischaemic injury [29]. Using an animal model, Darby et al. showed a sustained reduction in renal cortical and medullary oxygenation in rats which were subjected to 1 h of normothermic cardiopulmonary bypass with target Hb concentrations of 65 g/L [30]. Lastly, blood transfusions in patients with AKI receiving RRT have not been shown to have an impact on 90-day mortality or other patient-centred outcomes [31].

To the best of our knowledge, the question whether anaemia affects the chances of renal recovery has only been explored once before in a retrospective study of 211 hospitalised patients [32]. The authors defined AKI by the following: 0.5 mg/dL increase in serum creatinine if the baseline serum creatinine was ≤1.9 mg/dL; 1.0 mg/dL increase for baseline serum creatinine 2.0–4.9 mg/dL; 1.5 mg/dL increase if baseline serum creatinine was ≥5.0 mg/dL. Renal recovery was defined by complete or near complete return of the creatinine to baseline, 50 % reduction in creatinine from the peak creatinine, or discontinuation of RRT. The study showed that neither renal recovery nor survival differed among patients with mild versus severe anaemia as measured by fall in Hb or nadir Hb. Although we defined AKI differently, and focussed on progression from AKI stage I to AKI stage III versus non-progression, our study reaches a similar conclusion. Using two separate haemoglobin cut-offs, we found that the risk of progression was not increased in patients with AKI I and Hb <80 or Hb <100 g/L. The previously demonstrated association between DO_2_I and AKI progression [6] was predominantly determined by underlying cardiac index but not Hb or SaO_2_. Of note, we previously also found that once AKI was established, attempts to increase a low DO_2_I did not prevent progression to AKI III [6] and excessive fluid administration resulting in fluid accumulation was associated with an increased risk of progression [7].

Given the high prevalence of anaemia in critically ill patients, restrictive transfusion strategies and the high incidence of AKI in critically ill patients, these results are important. Nevertheless, some unanswered questions remain. Firstly, we retrospectively analysed the data of critically ill patients with AKI only in those where haemodynamic monitoring was considered to be required. Whether anaemia affects renal recovery in patients who are less sick is not clear. Second, we defined AKI by the creatinine and RRT criteria of the AKIN classification only [25]. It is possible that the association between anaemia and progression to AKI III would have been different if we had also used the urine criteria to identify patients with AKI. Third, we were not able to explore whether blood transfusion was a risk factor for progression of AKI. A recent retrospective study in patients with moderate anaemia showed an increased incidence of AKI in those who were transfused [33]. We cannot exclude that the theoretical benefits of a higher Hb were counteracted by adverse effects from blood transfusions. Fourth, we did not determine the aetiology of anaemia and explore whether it was acute or chronic. Also, we did not correct Hb levels for fluid accumulation but note that there was no difference in cumulative fluid balance on the day of AKI I. Fifth, we acknowledge that our results may contradict those studies which showed less AKI in cardiac surgery patients who had received EPO treatment preoperatively [23, 24]. However, it is possible that these effects were not simply due to correction of Hb but also prevention of blood transfusion and other EPO-related organ protective effects, including inhibition of apoptosis [34]. Finally, we analysed data from 2007 to 2009 which included relatively few patients with Hb <80 g/L. The cohort may have been too small to detect a potential association between severe anaemia and AKI progression.

## Conclusions

Our study shows that, whilst a low cardiac index on the first day of AKI was associated with progression to more severe AKI, anaemia was not. Unanswered questions remain and further investigation is warranted. Until such time, clinicians should prioritise optimisation of cardiac index to reduce the risk of progression of AKI, but there is no evidence that a higher Hb confers protection.

## Key messages

- In critically ill patients with acute kidney injury (AKI) stage I, the mean haemoglobin on the first day of AKI was 96 g/L.
- There was no difference in the haemoglobin concentration of patients who progressed to AKI III and those who did not progress.
- Pre-existing heart disease, need for mechanical ventilation, arterial lactate, SOFA score, central venous pressure and cardiac index on the first day of AKI stage I were independently associated with an increased risk of progression from AKI stage I to AKI stage III but haemoglobin was not.

## Abbreviations

AKI: Acute kidney injury
AKIN: Acute Kidney Injury Network
APACHE: Acute Physiology and Chronic Health Evaluation
AUC: Area under the curve
CAD: Coronary artery disease
CCF: Congestive cardiac failure
CKD: Chronic kidney disease
CVP: Central venous pressure
DO_2_I: Oxygen delivery index
EPO: Erythropoetin
Hb: Haemoglobin
ICU: Intensive care unit
IQR: Interquartile range
MAP: Mean arterial pressure
ROC: Receiver operating characteristic
RRT: Renal replacement therapy
SaO_2_: Arterial oxygen saturation
SD: Standard deviation
SOFA: Sequential Organ Failure Assessment
